# Chemical Composition of *Ailanthus altissima* (Mill.) Swingle Methanolic Leaf Extracts and Assessment of Their Antibacterial Activity through Oxidative Stress Induction

**DOI:** 10.3390/antibiotics12081253

**Published:** 2023-07-29

**Authors:** Halima Boukhibar, Aicha Laouani, Soraya Naila Touzout, Rawaf Alenazy, Mohammed Alqasmi, Yaseen Bokhari, khaled Saguem, Mossadok Ben-Attia, Safia El-Bok, Abderrahmen Merghni

**Affiliations:** 1 Laboratory of Biodiversity, Biotechnologies and Climate Change (LR11/ES09), Faculty of Sciences of Tunis, University of Tunis El-Manar, Tunis 2092, Tunisia; boukhibar.saida@gmail.com (H.B.); sorayatouzout@gmail.com (S.N.T.); safia.elbok@fst.utm.tn (S.E.-B.); 2 Laboratory of Metabolic Biophysics and Applied Pharmacology (LR12/ES02), Faculty of Medicine of Sousse, University of Sousse, Sousse 4002, Tunisia; aicha.laouani@famso.u-sousse.tn (A.L.);; 3 USCR Analytical Platform UHPLC-MS &Research in Medicine and Biology, Faculty of Medicine of Sousse, University of Sousse, Sousse 4002, Tunisia; 4 Department of Medical Laboratory, College of Applied Medical Sciences-Shaqra, Shaqra University, Shaqra 11961, Saudi Arabia; malqasmi@su.edu.sa; 5 College of Pharmacy, Alfaisal University, Riyadh 12714, Saudi Arabia; ybokhari@alfaisal.edu; 6 Environment Biomonitoring Laboratory (LR01/ES14), Faculty of Sciences of Bizerte, University of Carthage, Zarzouna 7021, Tunisia; mossadok.benattia@fsb.ucar.tn; 7 Laboratory of Antimicrobial Resistance LR99/ES09, Faculty of Medicine of Tunis, University of Tunis El Manar, Tunis 1006, Tunisia

**Keywords:** *A. altissima*, methanolic extracts, HPLC-DAD, antibacterial properties, oxidative stress, pathogenic bacteria

## Abstract

The present study was conducted to investigate the chemical composition of *Ailanthus altissima* (Mill.) Swingle methanolic leaf extracts from geographically distinct regions and to assess their antimicrobial properties along with their ability to induce oxidative stress. The HPLC-DAD analysis revealed the presence of phenolic acids and flavonoids including chlorogenic acid, gallic acid, synapic acid, p-coumaric acid, apigenin, hyperoside, isoamnétine-3-O-beta-D-glucotrioside, quercetin, and isoquercetin in various amounts depending on the origin of tested extracts. The assessment of antibacterial activity showed the effectiveness of the *A. altissima* extracts particularly against Gram-positive bacteria, with inhibition zone diameters reaching 14 ± 1 mm and minimum inhibitory concentrations ranging from 4 to 72.2 mg/mL. These bioactive substances also exhibited strong antibiofilm activity with an eradication percentage reaching 67.07%. Furthermore, they increased ROS production to levels two to five times higher than the control group, altered the membrane integrity and caused lipid peroxidation with MDA production exceeding 2.5 µmol/mg protein in the Gram-positive and Gram-negative strains. A decrease in the levels of the antioxidant enzymes SOD and CAT was also observed, indicating an impairment of the bacterial response to the oxidative stress caused by the tested extracts. These findings highlight the antibacterial properties of *A. altissima* leaf extracts depending on their origins and promote their exploitation and application in the agro-food and pharmaceutical sectors.

## 1. Introduction

The ongoing increase in bacterial resistance poses a global threat to public health resulting in financial pressures placed on patients, insurance companies and healthcare systems [1]. The World Health Organization released a list of problematic resistant bacteria grouped under the acronyms ESKAPE (*E. faecium*, *S. aureus*, *K. pneumoniae*, *A. baumannii*, *P. aeruginosa*, *Enterobacter* sp.) and ESCAPE (*E. faecium*, *S. aureus*, *C. difficile*, *A. baumannii*, *P. aeruginosa*, Enterobacteriaceae) [2]. The crisis of antibiotic resistance is attributed to the overuse and misuse of these medications in addition to the lack of new drug development [3].

Bacteria have developed sophisticated mechanisms to evade antibiotic-induced killing, which can be categorized as intrinsic resistance (related to inherent structural or functional characteristics of bacterial species) or acquired resistance (resulting from mutations in chromosomal genes or the acquisition of external genetic determinants) [4]. Furthermore, the problem of antibiotic resistance is exacerbated by the microbial biofilms, which are microbial communities of microorganisms adhered to surfaces and shielded by a matrix of extracellular polymeric substances [5]. Theses biofilms display an increased tolerance to antibiotics and other chemical inhibitors [5].

Natural products are experiencing a resurgence of interest and offer potential solutions to deal with the healthcare crises, particularly those related to the infectious diseases [6]. Much research has focused on the efficacy of photochemical antibacterial products as potential alternatives to synthetic antibiotics. The antimicrobial activity of plant extracts is of particular interest as it encourages the idea of using alternative bioactive substances [7]. For instance, *Ailanthus altissima* (Mill.) Swingle, which is an exotic tree belonging to the *Simaroubaceae* family and native to China, has been introduced and allowed to naturally grow in various regions worldwide. Methanolic extracts of *A. altissima* demonstrated a promising antibacterial activity in different studies. These extracts effectively combat both Gram-positive and Gram-negative bacteria [7].

One of the targeted modes of action of plant extracts is the induction of oxidative stress in pathogenic bacteria through the enhancement of reactive oxygen species (ROS) generation [8]. ROS are considered as an intracellular apoptotic factor in bacteria as their production leads to the oxidation of proteins, lipids, DNA and membrane degradation [9]. This antagonistic activity is probably attributed to the presence of specific compounds in the plants extracts such as gallic acid, rutin, hyperoside, epicatechin, luteolin derivatives, and apigenin derivatives which have been identified in the literature to potentially possess antibacterial activities [10,11,12,13,14].

The significance of oxidative stress in pathogenic bacteria highlights its role as a crucial factor in developing new antibacterial agents with potential antibacterial effects [15]. Therefore, the current study aims to analyse the methanolic composition of *A. altissima* leaves and investigate their antibacterial and antibiofilm activities, along with the induction of oxidative stress in Gram-positive and Gram-negative bacteria.

## 2. Results

### 2.1. HPLC-DAD Analysis for Phenolic Compound Characterization

The analysis using HPLC-DAD indicated the presence of phenolic acids and flavonoids in the leaves of *A. altissima* from four regions. The detected phenolic acids (Table 1) included gallic acid (Peak 1; Rt: 2.862 min), chlorogenic acid (Peak 2; Rt: 6.055), p-coumaric acid (Peak 3; Rt: 11.433), and sinapic acid (Peak 4; RT: 14.103). Isorhamnetin 3-O-beta-D-glucotrioside, which is a phenolic compound belonging to the flavonoid family, was also detected (Peak 5; Rt: 15.516). Hyperoside (Peak 6; Rt: 16.64), Isoquercetin (Peak 7; Rt: 17.70), quercetin (Peak 8: Rt: 30), and apigenin (Peak 9: 40.985) were identified as well.

**Figure 1:**
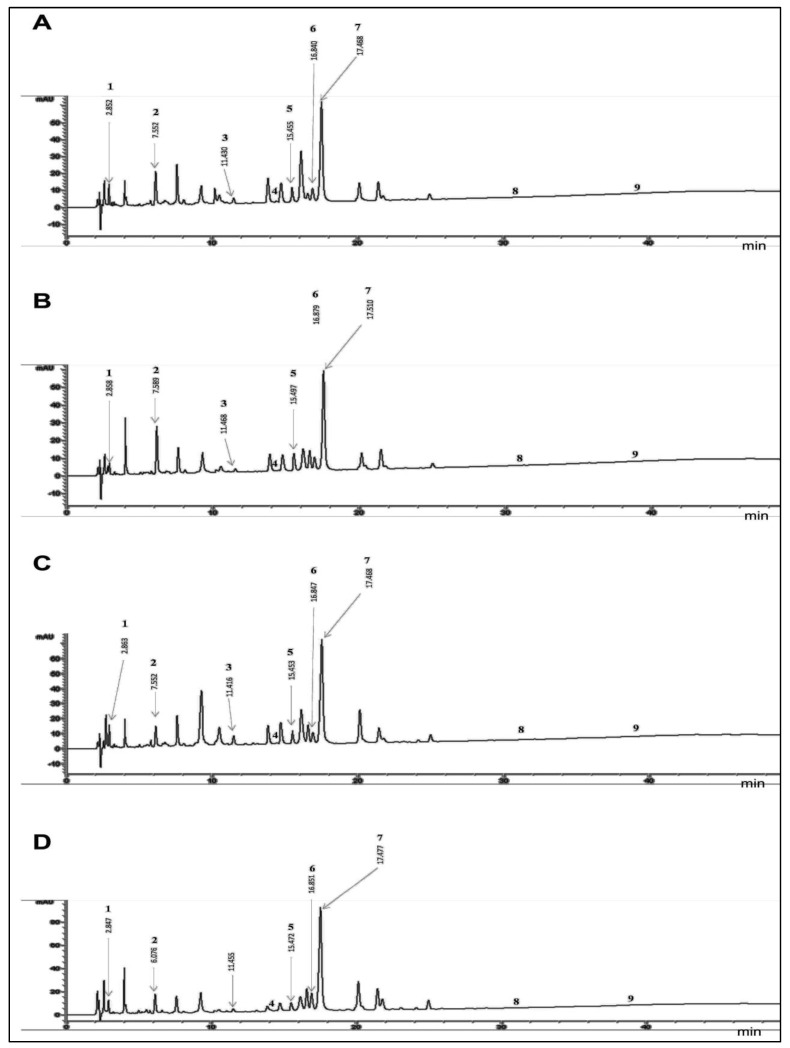
HPLC-DAD chromatograms of methanolic extracts of *A. altissima* leaves from different Algerian and Tunisian regions: (**A**) Bizerte, (**B**) Sousse, (**C**) Tlemcen, (**D**) Blida. Peak identifications were confirmed by retention times (Rt) and as deduced from standard compounds: (1) gallic acid, (2) chlorogenic acid, (3) p−coumaric acid, (4) synapic acid, (5) isorhamnetin 3−o−beta−d−glucotrioside, (6) hyperoside, (7) isoquercetin, (8) quercetin, (9) apeginin.

The base peak chromatogram of the methanolic extracts is presented in Figure 1. The compounds were labeled according to their elution order. A qualitative similarity was observed in the nine identified compounds across the four regions. In terms of quantity, isoquercetin was the major compound followed by chlorogenic acid, isorhamnetin 3-O-beta-D-glucotrioside, hyperoside, p-coumaric acid, and gallic acid. Quercetin, sinapic acid, and apigenin were present in smaller quantities in the plant.

### 2.2. Antibacterial Activity

#### 2.2.1. Disc Diffusion Test

The obtained results from the disc diffusion assay are presented in Table 2. *A. altissima* methanolic leaf extracts showed an effective antibacterial activity, but this activity varied depending on the tested bacteria. The most significant effect (*p* < 0.05) was recorded against Gram-positive bacteria *S. aureus* (14.00 ± 10 mm) and *S. epidermidis* (13.00 ± 10 mm) while it was less noticeable in Gram-negative bacteria *E. coli* (5.66 ± 0.57 mm) and *P. aeruginosa* (6.66 ± 0.57 mm).

#### 2.2.2. Determination of Minimum Inhibitory and Minimum Bactericidal Concentrations

The antibacterial activity expressed as MICs and MBCs was mostly aligned with the observed inhibition diameters. Extracts that produced larger inhibition zones usually had lower MIC values for the corresponding strains. In other words, the larger the inhibition zone, the lower the minimum concentration required to inhibit bacterial growth. This was recorded against the *S. aureus* strain with a concentration of 0.5 mg/mL (Table 3).

### 2.3. Antibiofilm Activity

The crystal violet method was used to quantitatively evaluate the antibiofilm effects of the tested extracts. The obtained results are shown in Table 4.

Our findings revealed a positive correlation between biofilm eradication effects of the extract, and the used concentration. Specifically, samples treated with a concentration of 4 × MIC showed the highest rates of biofilm eradication (*p* < 0.001). For example, in the *S. aureus* strain, the eradication percentages ranged from 17.62% to 67.07%. Similarly, in *S. epidermidis*, the eradication rates ranged from 15.19% to 77.03%. In *P. aeruginosa*, the percentages varied from 12.13% to 61.1%, and in *E. coli*, they ranged from 9.05% to 66.55%.

### 2.4. Cell Membrane Integrity

**Figure 2:**
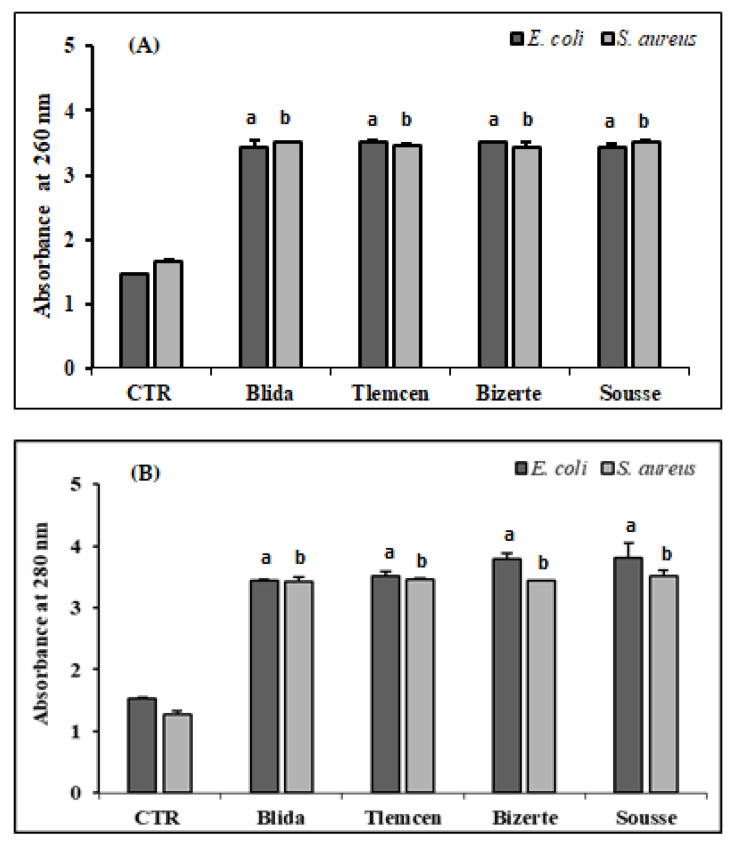
Release of cell content of *S. aureus* ATCC 25923 and *E.coli* ATCC 25922 evaluated by measuring and quantifying the absorbance at 260 nm (**A**) and 280 nm (**B**) treated with methanolic extracts of *A. altissima* from different regions. Control (CTR): bacteria were untreated with plant extracts. Results are expressed as mean ± SD. Absorbance values are expressed as mean ± SD. Comparison of absorbance between CTR and *E. coli* or *S. aureus* treated with leaf extracts from each studied region were performed using the unpaired two-tailed Student’s *t*-test. Significant differences among tested groups are indicated by different letters. ^a^ *p* < 0.001 *E. coli* values significantly different from CTR and ^b^ *p* < 0.001 *S. aureus* values significantly different from CTR.

The results presented in Figure 2 showed a notable rise in the absorbance of the supernatant because of the liberation of nucleic acids (260 nm) and proteins at (280 nm) from *S. aureus* and *E. coli* cultures compared to the control. The methanolic extracts in their 2 × MIC showed an increase of 2.1 until 2.4 times at 260 nm and 2.5 to 2.7 times at 280 nm (*p* < 0.001).

### 2.5. ROS Generation

**Figure 3:**
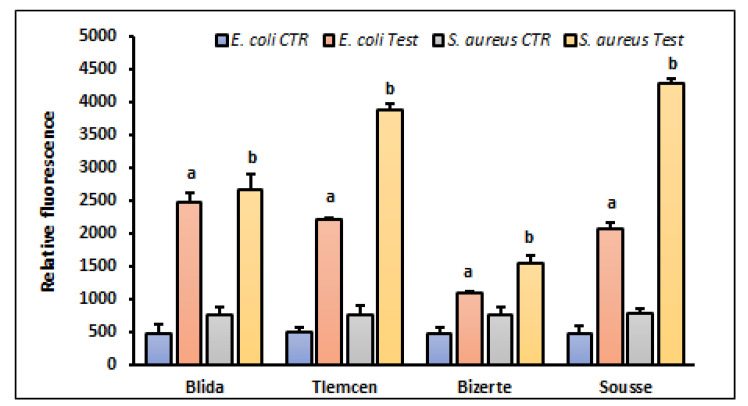
ROS production by *E. coli* and *S. aureus* strains after treatment with the leaf extracts of *A. altissima* from different regions. Control (CTR): bacteria were untreated with plant extracts. Results are expressed as mean ± SD. Comparisons of fluorescence intensity between CTR and *E. coli* or *S. aureus* treated with leaf extracts from each studied region were performed using the unpaired two-tailed Student’s *t*-test. Significant differences among tested groups are indicated by different letters. ^a^ *p* < 0.001 indicates *E. coli* values are significantly different from CTR and ^b^ *p* < 0.001 indicates *S. aureus* values are significantly different from CTR.

The generation of ROS in control cells was low, while the enhanced ROS production was noticed in our samples ranging from two to five times higher than the control group (*p* < 0.001), although the divergence between sources was not highly remarkable (Figure 3).

### 2.6. Malondialdehyde (MDA) Production

**Figure 4:**
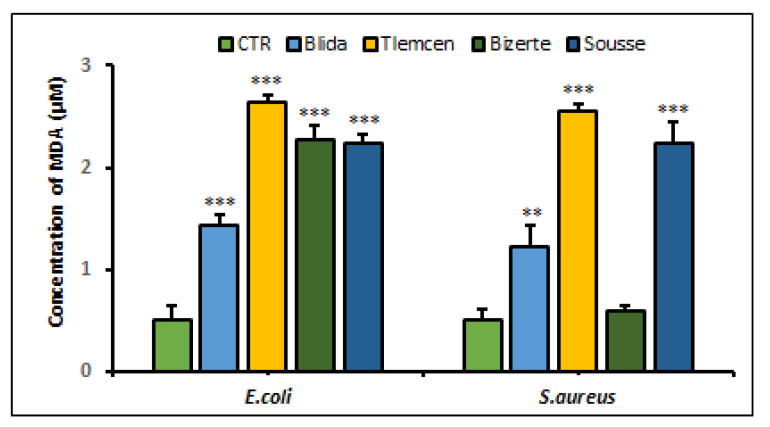
Quantification of MDA production by *E. coli* ATCC 25922 and *S. aureus* ATCC 25923 after 24 h of treatment with extracts of *A. altissima* from different studied regions. Control (CTR): bacteria were untreated with plant extracts. MDA levels are expressed as mean ± SD. Comparison of MDA rates between CTR and *E. coli* or *S. aureus* treated with leaf extracts from each studied region were performed using the unpaired two-tailed Student’s *t*-test. ** *p* < 0.01 and *** *p* < 0.001 significantly different from CTR.

In comparison with the control of 0.51 ± 0.14 µmol MDA/mg protein for *E. coli* ATCC 25922 and 0.5 ± 0.13 for *S. aureus* ATCC 25923, the concentration of MDA in these treated cultures increased (Figure 4). For *S. aureus*, the highest increase in MDA levels was observed in the Sousse and Tlemcen extracts, which were five times higher than the control, while the highest increase in *E. coli* was observed in the Tlemcen extract, which was two times higher than the control (*p* < 0.001). These results corroborate the evident presence of oxidative stress due to the ability of this synthetic radical to generate free radicals that cause lipid oxidation.

### 2.7. SOD Activity

**Figure 5:**
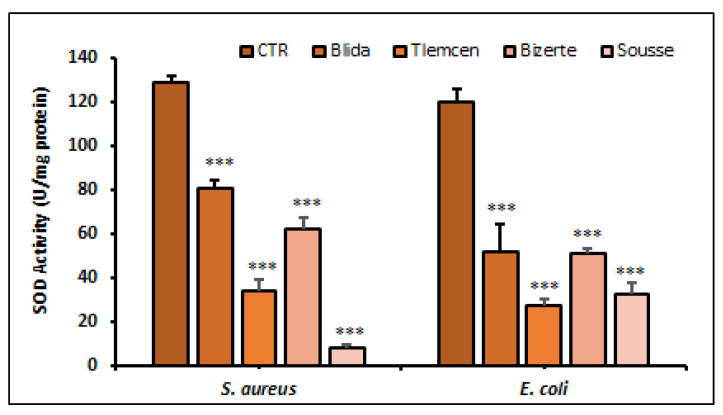
Determination of SOD activity (SOD/mg protein) in *E. coli* and *S. aureus* in the presence of *A. altissima* extracts. Control (CTR): bacteria were untreated with plant extracts. Data reported as mean ± SD. SOD activity comparison between CTR and *E. coli* or *S. aureus* treated with leaves extracts from each studied region were performed using the unpaired two-tailed Student’s *t*-test. *** *p* < 0.001 significantly different from CTR.

The extracts resulted in a significant decrease in the level of SOD in the bacterial strains compared to the control group including *S. aureus* ATCC 25923 (128.78 ± 0.3 SOD/mg protein) and *E. coli* (120.23 ± 0.6 SOD/mg protein). Specifically, the extracts from Tlemcen and Sousse showed a significant depletion of SOD in *E. coli* ATCC 25922, with values of (27.01 ± 0.29) and (32.65 ± 0.46), respectively (Figure 5). In regard to *S. aureus* ATCC 25923, the SOD levels were (33.81 ± 0.53) and (7.86 ± 0.17) in the same aforementioned sources, respectively (*p* < 0.001).

### 2.8. Catalase Activity

**Figure 6:**
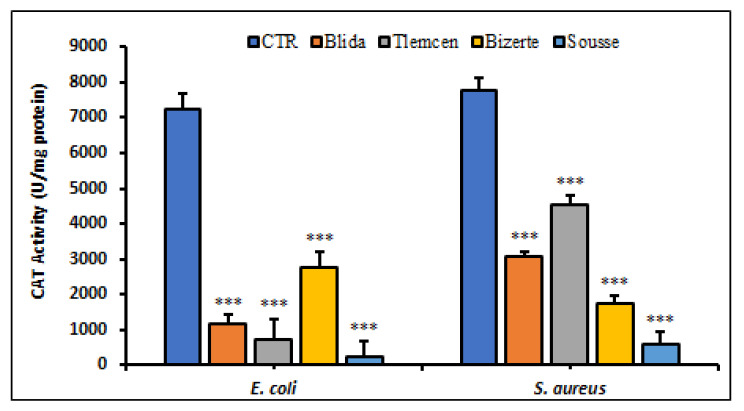
Determination of CAT activity (U/mg protein) in *E. coli* and *S. aureus* in the presence of *A. altissima* extracts. Control (CTR): bacteria were untreated with plant extracts. Data reported as mean ± SD. CAT activity comparison between CTR and *E. coli* or *S. aureus* treated with leaf extracts from each studied region were performed using the unpaired two-tailed Student’s *t*-test. *** *p* < 0.001 significantly different from CTR.

The results revealed a significant reduction in catalase activity (*p* < 0.001). In *E. coli* ATCC 25922, there was a decrease of 2 to 98 times compared to the control group with the lowest value observed in the samples from Sousse and Tlemcen. In *S. aureus* ATCC 25923, we observed a reduction in catalase production ranging from 13 to 17 times (Figure 6). The decrease in catalase activity caused by our extracts may lead to an increase in H_2_O_2_ levels, which could induce oxidative stress toxicity in the bacterial cells.

## 3. Discussion

Extensive research using diverse analytical techniques has been conducted on several parts of *A. altissima* resulting in the identification of phenolic acids and their derivatives. Therefore, the presence of benzoic acid, ferulic acid, vanillic acid, gallic acid and caffeic acid was reported [16]. Furthermore, it has previously been shown that there is an abundance of chlorogenic and caffeic acid in both dried and fresh leaves of *A. altissima*, which is in agreement with our findings. Anterior investigations expanded the knowledge of phenolic acids in this plant revealing the presence of additional compounds such as ellagic acid, p-coumaric acid, salicylic acid, syringic acid, protocatechuic acid and p-hydroxybenzoic acid, and other related compounds [17]. The observed differences between our samples and previous studies could be attributed to several factors including the geographic origin of the samples, inter-species variations and the specific analytical conditions employed [16,17,18]. According to a recent investigation of the phytochemical profiles of *A. altissima*, our study has further expanded the knowledge of the chemical composition of this plant, highlighting the new phenolic compounds, namely, synapic acid and isorhamnetin 3-O-beta-D-glucotrioside [16].

Following the chemical composition analyses, we firstly investigated the antibacterial activity of the methanolic leaf extract of *A. altissima* which is commonly used to efficiently target the phenolic compounds and secondary metabolites [19]. As for the obtained results from the disc diffusion method, all the tested extracts were found to be active against Gram-positive strains, inducing an inhibition zone of at least 10 mm [20]. The antibacterial activity of the extracts expressed as MICs and MBCs was generally aligned with the observed inhibition diameters since the extracts that produced larger inhibition zones usually had lower MIC values in the corresponding strains tested. On the other hand, we noticed that *A. altissima* extracts were less active against *E. coli* and *P. aeruginosa*. These differences can be explained by the presence of an outer membrane in Gram-negative bacteria which is composed of lipoproteins and lipopolysaccharides, making them less susceptible to the active components from the plant extracts. However, Gram-positive bacteria have a less restrictive membrane making them more sensitive to the antibacterial effects of the extracts [21]. A previous finding conducted by Rahman et al., Ref. [22] revealed that methanolic extracts derived from *A. altissima* leaves exhibited higher efficacy against Gram-positive bacteria, including *L. monocytogenes*, *S. aureus* and *B. subtilis*. However, based on our findings, theses extracts were found to be ineffective against Gram-negative bacteria such as *E. coli*, *S. enteritidis*, and *S. typhimurium*. The antibacterial efficacy of this plant could be attributed to the presence of specific compounds in the leaf extracts such as gallic acid, rutin, hyperoside, epicatechin, luteolin and apigenin derivatives. These compounds have been identified in the literature for their potent antibacterial effects [10,11,12,13,14].

In regard to antibiofilm activity, our findings revealed a positive correlation between the biofilm eradication effects of the extracts and the used concentration. Specifically, the samples treated with a concentration of MIC × 4 showed the highest rates of biofilm eradication. It has previously been demonstrated that quercetin, which is major component present in our studied extracts, has notably reduced the biofilm production of *E. coli* [23]. Additionally, this same molecule exhibited significant antibiofilm effects on the *P. aeruginosa* PAO1 strain [24]. Inhibitory effects of quercetin have also been reported against microbial biofilm of clinical isolates [25] and resistant strains like methicillin-resistant *S. aureus* (MRSA) and vancomycin-resistant *S. aureus* (VRSA) strains [26]. Furthermore, the chemical compound hyperoside, which is a flavonoid present in *A. altissima*, was found to be active against the *P. aeruginosa* biofilm [27]. In our study, it is worth noting that samples from the Sousse and Tlemcen regions showed relatively higher rates of eradication compared to other samples. This observation could be attributed to the specific climatic conditions of these regions. Indeed, the plants grown in environments subjected to significant environmental stress tend to activate their defense system and stimulate the biosynthesis of secondary metabolites which are responsible for their antibacterial properties [16].

Since the bacterial membrane is made up of a phospholipid bilayer, assessing cell leakage serves as an indicator of both membrane permeability and the integrity of bacterial cells [9]. Our results showed a notable rise in the absorbance of the supernatant because of the liberation of nucleic acids and proteins from the treated *S. aureus* and *E. coli* cultures compared to the control. It was reported that phenolic compounds present in the plant’s methanolic extract affects the porosity of bacterial cell membranes by altering intracellular mechanisms. These compounds interact with distinct functional groups of enzymes leading to a disintegration of the cell membrane [28]. Moreover, the phenolic compounds have the ability to interrupt the bacterial membrane integrity, leading to the leakage of essential intracellular constituents [29]. According to Sanchez et al., natural substances induce the hyperpolarization of bacteria which results in membrane damage [30]. A previous study reported that the exposure of *S. aureus* to a different phenolic compound, specifically chlorogenic acid, results in alterations of the cell membrane [31]. Due to the significant presence of this compound in our plant, it provides a plausible explanation for the notable increase in damage to the bacterial cell membrane of this strain.

One of the modes of action of phenolic compounds is the induction of oxidative stress in the bacterial cell as a result of increased reactive oxygen species (ROS) generation [32]. Our results revealed the enhanced ROS production of two to five times higher than the control of the Gram-positive and Gram-negative strains treated with each extract, without significant differences between the samples collected from different origins. ROS are recognized as internal inducers of apoptosis in bacteria because of their high production in cell cytoplasm which results in inducing the oxidation of proteins, lipids, and DNA [9]. It is worth mentioning that the *A. altissima* extracts enhanced the formation ROS in the tested bacterial strains which can lead to the destruction of iron–sulfur clusters resulting in the liberation of ferrous ions [8]. In the Fenton reaction, this iron has the ability to interact with hydrogen peroxide leading to a chain reaction that generates hydroxyl radicals capable of directly damaging DNA, lipids, and proteins [33]. The membrane lipids are responsible for the structure and function of membranes depending on their composition and arrangement. Lipids have the ability to alter the physical characteristics of membranes such as fluidity, rigidity, and permeability. These biophysical characteristics directly influence the cellular processes including the transport of molecules across the membrane, cell communication and signal transmission [34]. Lipid peroxidation is one of the side effects of high ROS production in cells. We quantified a byproduct of this process which is called malondialdehyde (MDA) as it is extensively utilized as a reliable biomarker in practical applications to assess the lipid peroxidation of fatty acids as a result of its high reactivity with thiobarbituric acid (TBA) [35,36]. When a comparison is made with the control group, the concentration of MDA in these treated cultures increased regardless of the origin of the used extract. Concerning *S. aureus*, the highest increase in MDA levels was observed in the extracts collected from Sousse and Tlemcen, while the highest increase in *E. coli* was observed in the Tlemcen extract. These results confirm the evident presence of oxidative stress due to the ability of this synthetic radical to generate free radicals that cause lipid oxidation [37]. This finding highlights the consistency with previous studies and confirms that the increase in lipid peroxidation results from the heightened production of ROS in the bacteria exposed to a methanolic extract of *A. altissima*.

ROS are highly reactive and significantly toxic in biological systems as they attack various macromolecules such as proteins, polyunsaturated lipids, and nucleic acids [38]. To deal with this form of stress, superoxide dismutase (SOD) represents the first line of defense of bacterial cells against ROS attack by catalyzing the conversion of superoxide into hydrogen peroxide. In our study, the significant decrease in SOD levels which was observed after treating the bacteria with *A. altissima* extracts indicates an impairment of bacterial response to oxidative stress. These results are consistent with the notion that superoxide dismutase plays a crucial role in protecting cells from ROS damage. When SOD levels are reduced, cells become more vulnerable to the harmful effects of ROS leading to an increase in the level of reactive oxygen species and cellular deterioration [39]. For instance, the methanolic extract of *A. paniculata* caused a significant decrease in SOD activity in the treated *S. aureus* strain, when compared with untreated cells [40]. Following the treatment of *E. coli* with PEBT (2-phenylethynylbutyltellurium), a decreased level of SOD activity was recorded [41]. All these observations highlight the crucial role of SOD in bacterial protection against oxidative stress and qualifies *A. altissima* extracts as a suitable candidate for new therapeutic agents targeting bacterial tolerance to oxidants. Catalase enzyme plays an essential role in detoxifying toxic H_2_O_2_ facilitating its conversion into water and O_2_ [42]. Our results revealed a significant reduction in catalase activity in both pathogenic strains of *E. coli* and *S. aureus* treated with a methanolic extract of *A. altissima*. Only one type of catalase, called Kat-A, has been identified in the *S. aureus* ATCC 25923 strain. On the other hand, *E. coli* has two catalases, katE and katG, which are also involved in hydrogen peroxide detoxification. However, the impact of these catalases on bacterial virulence is not clearly established due to the existence of compensatory mechanisms that may mitigate the effects of their reduced activity [42]. In other studies, similar results have been observed with other phytochemical compounds such as silibin which reduced catalase activity and induced toxicity in *S. aureus*. Previous studies have also shown a decrease in catalase activity after being treated with allylpyrocatechol [43] and *Leonurus cardiaca* [44] in *S. aureus*. These results highlight the importance of catalase in protecting against oxidative stress and underline the need to develop new therapeutic strategies for targeting H_2_O_2_ detoxification and oxidative stress in bacterial pathogens.

## 4. Materials and Methods

### 4.1. Collection and Preparation of Plant Material

*A. altissima* leaves used in this study were collected in 2019 from different regions of Algeria (Blida and Tlemcen) as well as Tunisia (Sousse and Bizerte). Taxonomic identification of the studied plant was carried out by the help of the expert Mounir KASRI at INAT (Carthage University). A voucher specimen of leaves of this plant has been deposited in the herbarium of our laboratory located in the Department of Biology, Faculty of Sciences of Tunis (Tunisia) under the following registration numbers: A.al-Biz/19-001, A.al-Sou/19-002, A.al-Tlem/19-003 and A.al-Bli/19-004. *A. altissima* Leaves were subjected to natural air drying at room temperature for a period of one week, then they were powdered finely and sieved through a mesh sieve with a pore size of 0.5 mm to obtain particles of uniform size. Subsequently, the obtained powder was stored for subsequent investigations.

### 4.2. Preparation of Plant Extracts

The samples underwent a maceration in a solution of 80% methanol (methanol/water, 80/20 *v*/*v*). The obtained mixture was subsequently filtered using a Wattman #1 filter paper from Bärenstein, Germany. The solvent was evaporated using a rotary evaporator (Buchi Rotavapor R-215, Marshall Scientific, Tide Mill Rd, Hampton, VA, USA). The remaining residue was then subjected to analysis to determine its phytochemical composition and conduct further biological investigations.

### 4.3. HPLC-DAD Analyses

The chromatographic analyses of methanolic extracts from different regions of Algeria (Blida and Tlemcen) as well as Tunisia (Sousse and Bizerte) were performed using an HPLC (high performance liquid chromatography) device of the Agilent 1200 type controlled by a computer. For the separation, the Kinetex Evo C18 analytical column was used at room temperature. The injection volume was 20 uL and peaks were monitored at 254 nm. Samples were filtered through at 0.22 um filter before injection. Mobile phase A was formed by 99% water and 1% formic acid; mobile phase B was formed by a combination of acetonitrile and formic acid (1%) with a flow rate of 1 mL/min. The chromatographic conditions were as follows: 90% A, 10% B (0 min), 80% A, 20% B (20 min), 75% A, 25% B (30 min), 65% A, 35% B (40 min) and 90% A 10% B (50 min). Under these conditions, the pump generated a pressure of approximately 150 bars. The measurement of optical density ensures spectrophotometric detection of the analyses detected at a fixed wavelength of 254 nm. The identification of *Ailanthus* phenolic compounds was conducted by comparing their retention time and UV spectra with available authentic standards. The quantification was measured by comparing the area of the peak of interest with that obtained in a chromatogram of the standard with a known concentration.

### 4.4. Bacterial Strain

To assess the antibacterial activity, we used two Gram-positive strains which are referred to as *Staphylococcus aureus* ATCC 25923 and *Staphylococcus epidermidis* ATCC 2059, in addition to two Gram-negative strains, *Escherichia coli* ATCC 25922 and *Pseudomonas aeruginosa* ATCC 27853). Prior to each use, the tested strains were sub-cultured twice from glycerol stocks onto a tryptic soy broth (TSB; Difco) and incubated at 37 °C for 24 h to ensure optimal growth.

### 4.5. Disc Diffusion Assay

The disk diffusion method as described earlier by Pérez et al., Ref. [45] was utilized to assess the antibacterial activity of methanolic extracts obtained from various sources of *A. altissima*. Sterile (MH) Mueller Hinton agar was prepared and dispensed into aseptic 9 cm agar plates, and then they were inoculated with bacterial suspensions having an optical density of approximately 0.5 McFarland by using a sterile cotton swab. Afterwards, the paper filter discs of 6 mm diameter were carefully set on the agar surface. These discs were subsequently saturated with 10 μL of methanolic extracts with concentrations of 300 and 150 mg/mL, dissolved in dimethyl sulfoxide. As a positive control, a standard antibiotic gentamicin (Thermo Scientific, Basingstoke, UK) was applied to the paper discs (10 µL/disc) and followed by an incubation at 37 °C for 24 h. The diameter of the zones of inhibition (DZI) surrounding each disc was estimated to evaluate the antibacterial activity. The experiment was conducted in triplicate to ensure the reliability and consistency of the desired results.

### 4.6. Determination of the Minimal Inhibitory and Bactericidal Concentrations

This test aims to confirm the values of the minimum inhibition concentration (MIC) and minimum bactericidal concentration (MBC). To achieve this, 96-well plates from Polylabo, Strasbourg, France were filled with 95 µL of Mueller–Hinton liquid medium. Subsequently, the initial well was supplemented with 100 µL of the highest concentration (250 mg/mL), and two-fold serial dilutions were performed in the remaining 10 wells. Finally, 5 µL of the inoculum from each strain was added resulting in a final volume of 200 µL. The negative control, which serves as a reference and does not contain any plant extract, was also included. The plates were placed in an incubator at a temperature of 37 °C for 24 h.

The minimum inhibition concentration (MIC) was identified as the lowest concentration of the sample where there is an absence of visible growth in comparison with the negative control. The determination of values related to the minimum bactericidal concentration (MBC) involved extracting 10 µL from each well that showed no observable growth and inoculating it into Mueller–Hinton (MH) agar. After incubating the agar plates at 37 °C for 24 h, bacterial counts were obtained through measurement. The absence of growth indicated the bactericidal action of the sample according to the method described by Conforti et al. in 2003 [46]. In addition to that, 10 µL of MTT solution containing formazan crystals was added to each well and followed by incubation at a temperature of 37 °C for 3 h. The living cells reduced the tetrazolium in the MTT solution to formazan, resulting in the formation of a purple-colored precipitations in the mitochondria. This precipitate helped in the visualization of bacterial growth [47].

### 4.7. Biofilm Formation

Suspensions of 200 µL of the four bacterial strains were put in the 96-well plates and were kept undisturbed for 24 h at 37 °C to develop a bacterial film [48]. After performing a wash, we applied a treatment consisting of methanolic extracts dissolved in DMSO and BHI broth (Bio-Rad, Marnes-la-Coquette, France) at concentrations of 1 × MIC, 2 × MIC, and 4 × MIC. The plates were then incubated for 24 h. Then, for each well, 100 µL of crystal violet was added and incubated for a period of 30 min. The quantification of bacterial biofilm biomass is performed using a microplate reader (D.E.E.D. Reader, Bio-Rad) at a wavelength of 570 nm. The percentage of biofilm eradication is calculated by assessing the reduction in biofilm biomass using the following formula: [(OD growth control − OD sample)/OD growth control] × 100. The wells with untreated bacteria served as a negative control.

### 4.8. Membrane Integrity

The control of membrane permeability and integrity has a substantial impact on the natural defense of bacteria. To assess the integrity of the bacterial membranes, bacterial suspensions were treated with a concentration of 2 × MIC and maintained at 37 °C for 2 h. Finally, the suspensions underwent centrifugation at 3000 rpm for 10 min. Using a spectrophotometer, the optical density of the supernatant was measured at a wavelength of 260 nm and then at 280 nm to quantify the membrane integrity [49].

### 4.9. Reactive Oxygen Species (ROS) Detection

The reactive oxygen species (ROS) generation in *E. coli* ATCC 25922 and *S. aureus* ATCC 25923 were detected after a treatment with *A. altissima* extracts was conducted using a peroxynitrite indicator, dichlorodihydro-fluorescein diacetate (DCFH-DA), from Sigma Aldrich, UK. DCFH-DA is capable of detecting a wide range of ROS, including nitric oxide and hydrogen peroxide. The aforementioned indicator was utilized to examine the generation of ROS in the bacterial strains subsequent to exposure to *A. altissima* extracts. Bacterial suspensions in the exponential phase (0.5 McFarland) were adjusted and treated with extracts at concentrations of 2 × MIC. This treatment was performed in the presence of DCFH-DA, with a concentration of 5 mM in 0.85% saline solution. The suspensions were then aerobically incubated at a temperature of 37 °C for a duration of 24 h. A bacterial culture without any treatment was used as a negative control. To measure DCFH-DA fluorescence emission, a Biotek FL 800 microtiter plate reader was used with an excitation wavelength of 485 nm. The fluorescence emission was detected at a wavelength of 525 nm [50].

### 4.10. Lipid Peroxidation

Malondialdehyde (MDA) is a naturally formed compound generated during the oxidation process of polyunsaturated fatty acids by reactive oxygen species (ROS). To measure MDA production, the optical density of bacterial culture was adjusted at 0.5 McFarland and then treated with a concentration equivalent to 2 times the minimum inhibitory concentration (MIC) for 24 h at 37 °C. Following the incubation period, the cells were lysed using an SDS lysis buffer, and thiobarbituric acid (TBA) was added. TBA reacts with MDA to form a complex. The suspension was subsequently immersed in a water bath for a duration of one hour, followed by cooling of the tubes on ice for five minutes to halt the reaction. Measurement of MDA production was performed using an Elisa reader (Biotek FL 800) at a wavelength of 532 nm [49].

### 4.11. Superoxide Dismutase (SOD) Activity

As described by Marklund [51], the enzymatic activity of Superoxide Dismutase (SOD) was determined. The principle was based on the capacity of SOD to restrain the oxidation of pyrogallol at 420 nm. To perform the assay, 2.85 mL of tris HCl, 0.1 mL of enzyme extract, and 25 µL of pyrogallol were combined, followed by a 30 s incubation period. A blank solution was prepared using 0.1 mL of water instead of the enzyme extract, following the same procedure. The absorbance of the solutions was identified at 420 nm. The following equation was utilized to calculate SOD activity:

% inhibition = (Abs blank − Abs test)/Abs test

### 4.12. Catalase (CAT) Activity

*S. aureus* ATCC 25923 and *E. coli* ATCC 25922 cultures were treated with an extract concentration equivalent to two times the minimum inhibitory concentration (MIC). After 24 h of incubation at 37 °C, the extract was subjected to centrifugation at 3000 rpm for 10 min, and the obtained pellet was rinsed with PBS. This washing process was repeated twice. To prepare the bacterial extracts for enzymatic assays, the pellet was diluted in a suspension containing 500 μL of cell lysate (consisting of 10 mM Tris-HCl, 1 mM EDTA, 0.1% Triton-X-100, and 150 mM NaCl). The mixture was then incubated for one hour at 37 °C, followed by centrifugation at 3000 rpm for 10 min to collect the supernatant for enzyme assays, as described by Martins et al. [52]. According to Acuña et al. [53], catalase (CAT) activity in bacterial extracts was determined using the following procedure: 20 mL of recovered bacterial lysate was placed in a quartz vessel containing 780 mL of catalase buffer with a pH = 7, along with 200 mL of hydrogen peroxide (H_2_O_2_) at a concentration of 20 mM. The optical density was measured at 240 nm at both t = 0 and t = 1 in the spectrometer, based on the position of the cells. A unit (U) of enzyme activity was determined as the amount of enzyme required to oxidize one mole of H_2_O_2_ in one second.

A computational study could be conducted to correlate the in vitro activities of *A. altissima* methanol leaf extracts against the major proteins of target bacteria with an in silico docking study, which can provide useful information on the molecular basis of the antibacterial activities and mode of action of the tested extracts. Furthermore, the cytotoxicity of *A. altissima* extracts could be investigated in various human cell lines for potential valorization of this plant.

## 5. Conclusions

Our findings have revealed that methanolic leaf extracts of *A. altissima* from different localities are characterized by the presence of various bioactive compounds including gallic acid, isoquercetin and hyperoside in multiple proportions. Additionally, the tested extracts exert strong antibacterial and antibiofilm activities against Gram-positive bacterial strains, affect the membrane’s integrity and increase ROS production which results in lipid peroxidation as well as the inhibition of antioxidant enzyme activity. These results significantly expand our understanding of the advantageous potentialities of *A. altissima* extracts, which could be valorized for various applications aiming to fight against pathogenic bacteria.

## Figures and Tables

**Table 1:** Phenolic compounds and retention time (Rt) of *A. altissima* methanolic extracts from different regions identified and quantified by HPLC-DAD (µg/100 mL).

Peak	Rt (min)	Chemicals Compounds	Bizerte (µg/100 mL)	Sousse (µg/100 mL)	Tlemcen (µg/100 mL)	Blida (µg/100 mL)
1	2.862	Gallic acid	1688.22 ± 43.89 ^a,b,c^	551.69 ± 20.41 ^d,e^	1735.76 ± 48.60 ^f^	1182.25 ± 35.47
2	6.055	Chlorogenicacid	13,334.70 ± 346.71 ^a,b,c^	19,826.73 ± 654.28 ^d,e^	7758.35 ± 232.75 ^f^	5699.74 ± 159.59
3	11.433	p-Coumaric acid	1466.68 ± 38.13 ^a,b,c^	531.82 ± 19.68 ^d,e^	2308.28 ± 64.63 ^f^	862.04 ± 25.86
4	14.103	Synapicacid	Trace	Trace	25.86 ± 0.96 ^f^	218.20 ± 6.55
5	15.516	Isorhamnetin 3-O-Beta-D- Glucotrioside	55.98 ± 1.68 ^a,b,c^	47.72 ± 1.53	2901.24 ± 81.23 ^f^	1575.93 ± 50.42
6	16.64	Hyperoside	2217.77 ± 66.53 ^a,b,c^	1806.26 ± 50.58 ^d,e^	1725.02 ± 46.58 ^f^	2811.18 ± 73.09
7	17.70	Isoquercetin	12,137.55 ± 339.85 ^a,b,c^	10,400.04 ± 312.10 ^d,e^	14,393.95 ± 388,64 ^f^	14,783.70 ± 369.59
8	30.83	Quercetin	Trace	5.18 ± 0.21 ^d,e^	2.81 ± 0.13 ^f^	24.60 ± 0.98
9	40.985	Apigenin	Trace	3.79 ± 0.21 ^d,e^	3.86 ± 0.10 ^f^	6.01 ± 0.86

Values of phenolic compounds are represented as means ± SD of three measurements and are statistically compared by one-way ANOVA followed by Tukey’s multiple comparisons tests. ^a^ *p* < 0.001 significantly different from Sousse; ^b^ *p* < 0.001 significantly different from Tlemcen; ^c^ *p* < 0.001 significantly different from Blida; ^d^ *p* < 0.001 significantly different from Tlemcen; ^e^ *p* < 0.001 significantly different from Blida; and ^f^ *p* < 0.001 significantly different from Blida.

**Table 2:** Inhibition zones of different *A. altissima* extracts against pathogenic bacteria.

Origin	Concentration	*S. aureus*	*S. epidermidis*	*E. coli*	*P. aeruginosa*
Blida	150 (mg/mL)	10.33 ± 0.75	10.33 ± 0.57	7.66 ± 0.56	6.66 ± 0.57
	300 (mg/mL)	11.33 ± 0.76	10 ± 1.01	8.66 ± 0.58	9 ± 0.02 **
Tlemcen	150 (mg/mL)	10.33 ± 0.57	10.33 ± 0.58	6.66 ± 0.55	7.33 ± 0.56
	300 (mg/mL)	11 ± 1.02	11.66 ± 0.56 *	7.66 ± 0.54	9.33 ± 0.53 *
Bizerte	150 (mg/mL)	8 ± 0.03	10.33 ± 0.58	5.66 ± 0.56	7.33 ± 0.55
	300 (mg/mL)	9.6 ± 0.58 **	11.33 ± 0.56	7.66 ± 0.57	8.66 ± 0.57
Sousse	150 (mg/mL)	11.66 ± 0.56	11 ± 1.02	6.33 ± 0.54	8 ± 0.03
	300 (mg/mL)	14 ± 1.01 *	13 ± 1.03	7.33 ± 0.57	9.33 ± 0.58 *
GEN	10 (µg/mL)	24	24	25	22

Comparison between increasing concentrations of leaf extracts from each studied region was performed using the unpaired two-tailed Student’s *t*-test. * *p* < 0.05 and ** *p* < 0.01 significantly different from the 150 mg/mL concentration. GEN: gentamicin antibiotic.

**Table 3:** Antibacterial activity of *A. altissima* methanolic extract expressed as MIC and MBC values.

Origin	MIC/MBC	Bacterial Strains			
	(mg/mL)	*S. aureus*	*S. epidermidis*	*E. coli*	*P. aeruginosa*
Blida	MIC	4	72.25	31.25	31.25
	MBC	16	125	250	>250
Tlemen	MIC	16	8	8	16
	MBC	72.50	125	250	250
Bizerte	MIC	4	16	31.25	125
	MBC	16	>250	>250	>250
Sousse	MIC	4	16	31.25	72.50
	MBC	>250	>250	>250	>250

MIC: minimum inhibitory concentration; CMB: minimum bactericidal concentration.

**Table 4:** Percentages of biofilm eradication after treatment with various concentrations of *A. altissima* extracts.

Bacterial	Concentration	Eradication of Biofilm (%)			
Strains	(mg/mL)	Blida	Tlemcen	Bizerte	Sousse
	MIC	17.62 ± 2.87 ^a,b^	27.2 ± 9.68 ^a,b^	15.71 ± 5.70 ^a,b^	33.36 ± 3.82 ^a,b^
*S. aureus*	MIC × 2	35.64 ± 6.28 ^c^	57.59 ± 13.02	36.62 ± 8.85 ^c^	50.75 ± 1.41
	MIC × 4	46.63 ± 2.04	67.07 ± 12.07	55.58 ± 2.96	60.62 ± 10.24
	MIC	15.55 ± 4.20 ^a,b^	17.91 ± 8.37 ^b^	15.19 ± 8.90	23.88 ± 4.08 ^a,b^
*S. epidermidis*	MIC × 2	31.28 ± 2.67 ^c^	28.52 ± 10.89 ^c^	40.17 ± 2.95 ^c^	47.64 ± 13.24 ^c^
	MIC × 4	46.77 ± 5.46	48.73 ± 6.24	50.39 ± 3.36	77.03 ± 5.77
	MIC	17.73 ± 2.28 ^a,b^	32.66 ± 4.46 ^b^	9.05 ± 3.59	47.86 ± 1.21 ^a,b^
*E. coli*	MIC × 2	36.14 ± 0.55 ^c^	43.88 ± 6.65 ^c^	22.97 ± 1.82 ^c^	59.01 ±4.15 ^c^
	MIC × 4	50.43 ± 7.50	66.33 ± 3.04	33.37 ± 2.30	66.55 ± 1.47
	MIC	13.92 ± 1.76 ^a,b^	14.42 ± 6.09 ^b^	12.13 ± 2.75 ^a,b^	17.68 ± 3.61 ^a,b^
*P. aeruginosa*	MIC × 2	24.27 ± 0.87 ^c^	20.96 ± 7.05	26.79 ± 5.99 ^c^	25.73 ± 3.57 ^c^
	MIC × 4	41.39 ± 3.31	32.9 ± 6.53	56.03 ± 7.20	61.1 ± 8.61

Biofilm eradication values are represented as mean percentages ± SDs of three independent measurements and are statistically compared by one-way ANOVA followed by Tukey’s multiple comparisons tests. ^a^ *p* < 0.001 significantly different from MIC × 2; ^b^ *p* < 0.001 significantly different from MIC × 4 and ^c^ *p* < 0.001 significantly different from MIC × 4.

## Data Availability

Not applicable.
